# Targeting Drug Delivery to the Lungs by Inhalation

**DOI:** 10.1155/S0962935194000724

**Published:** 1994

**Authors:** C. O'Callaghan

**Affiliations:** 1 Department of Child Health School of Medicine University of Leicester Clinical Science Building, Leicester Royal Infirmary, PO Box 65 Leicester LE2 7LX UK

## Abstract

Most drugs targeted to the respiratory tract are used for their local action. For example, ephidrine for nasal decongestion, beta-2 agonists for bronchodilatation, and inhaled steroids to suppress the inflammation seen in asthmatic airways. Since the drug is delivered directly to its required site, only a small quantity is needed for an adequate therapeutic response, and consequently there is a low incidence of systemic side effects compared with oral or intravenous administration. More recently, it has become apparent that the lining of the respiratory tract, from nasal mucosa to airways and alveoli, may be used for the absorption of a drug for its systemic effect. This route of administration may be particularly attractive if it avoids the metabolic destruction encountered when some drugs are administered by alternative routes (for instance, peptides and proteins are rapidly destroyed by peptidases when Oven by the oral route). If there is a lack ofclinical response to an aerosolized drug, it is important to question whether the drug has failed or whether delivery to the site of action is inadequate. To deliver therapeutic agents by inhalation to the lower respiratory tract, inhaled drug particles must have appropriate aerodynamic characteristics. In addition, the anatomy and pathophysiology of the patient's respiratory tract, mode of inhalation through the inhaler, and the characteristics of the inhalational device itself, may significantly affect drug deposition.


Mediators of Inflammation 3, S31-33 (1994)

MOIA9

### Targeting drug delivery to the lungs by inhalation

C. O'Callaghan

Department of Child Health, School of Medicine, University of Leicester, Clinical Science Building, Leicester Royal Infirmary, PO Box 65, Leicester LE2 7LX, UK

**MOST drugs targeted to the respiratory tract are used for their local action. For example, ephidrine for nasal decongestion, beta-2 agonists for bronchodilatation, and inhaled steroids to suppress the inflammation seen in asthmatic airways. Since the drug is delivered directly to its required site, only a small quantity is needed for an adequate therapeutic response, and consequently there is a low incidence of systemic side effects compared with oral or intravenous administration. More recently, it has become apparent that the lining of the respiratory tract, from nasal mucosa to airways and alveoli, may be used for the absorption of a drug for its systemic effect. This route of administration may be particularly attractive if it avoids the metabolic destruction encountered when some drugs are administered by alternative routes (for instance, peptides and proteins are rapidly destroyed by peptidases when given by the oral route). If there is a lack of clinical response to an aerosolized drug, it is important to question whether the drug has failed or whether delivery to the site of action is inadequate. To deliver therapeutic agents by inhalation to the lower respiratory tract, inhaled drug particles must have appropriate aerodynamic characteristics. In addition, the anatomy and pathophysiology of the patient's respiratory tract, mode of inhalation through the inhaler, and the characteristics of the inhalational device itself, may significantly affect drug deposition.**

**Key words:** Aerosol particle size, Drug delivery devices, Inhalers, Lungs, Metered dose inhalers, Nebulized drug delivery, Targeting drugs

### The Importance of Aerosol Particle Size

The aerodynamic size of a particle is a measurement that takes into account the diameter and density of a particle, and is the most important factor determining the site of drug deposition in the lung. Drug particle size has been shown to be clinically important (for example, a bronchodilator administered in an aerosol of small particles (1.8 μm diameter) results in more effective bronchodilatation than when given in an aerosol of larger particles).

The amount of drug reaching the site of action usually dictates the therapeutic affect. Thus, measurement of particle size without reference to the amount of drug (mass) contained in the particles likely to reach the lungs can be misleading. For example, an aerosol of a drug solution, containing 1 000 1 μm particles and ten 10 μm particles sounds ideal for drug delivery. However, a 10 μm particle contains the same mass of drug as the 1 000 1 μm particles. Thus, as the 10 μm particles are likely to be filtered at the nose, only a small amount of drug is likely to reach the lung. If an aerosol of ultra fine particles is used, it needs to be much more concentrated or inhaled over a much longer period of time to deliver an equivalent mass of drug. This is very difficult in practice and an aerosol of 2–3 μm diameter particles is considered most practical for penetration into the lung and delivery of a sufficient mass of drug to the site of action.[1]

Care needs to be taken before the introduction of new aerosol formulations or drug delivery devices, to ensure that the medication has the best chance of reaching the airways. For example, steroids for nebulization are in a suspension. Individual, micronized, steroid particles of 2 μm diameter acquire a coating of the carrier solution during nebulization, resulting in a larger droplet. Thus, a nebulizer chamber with a baffle system that only allows particles of 2 μm or less to leave the chamber, will release virtually no steroid particles for inhalation. The ideal nebulizer must release droplets large enough to contain steroid particles, but small enough to reach the intrathoracic airways.

© 1994 Rapid Communications of Oxford Ltd

### Anatomy and Physiology

*The nose:* The ability of the nose to act as a filter is enhanced by its narrow slit-like passages and the turbulent flow of air in it. In adults, few particles larger than 10 μm (e.g. pollen grains) are able to penetrate the nose during breathing at rest, while most particles smaller than 2 μm (e.g. mould spores) by-pass the nose. Inhaled particles trapped by the nasal filter are cleared from the nose within 30 min by mucociliary clearance, and swallowed.

The majority of young children nose breathe at rest, and may inhale nebulized aerosols nasally when given by face mask. Nasal breathing results in lower lung deposition of aerosolized drug in adults, but little is known about this in infants. The absence of nasal hair in young children and the fact that the infant's head is larger with respect to its body than in adulthood may make nasal breathing less of a problem in infants than might be expected.

*The lungs:* As young children breathe tidally when given aerosolized medication, this may reduce deposition of drug in the lung peripheries, compared with older, more compliant patients, who inhale deeply and slowly. If their airways become narrowed by mucus, inflammation or obstruction, the linear velocity of air increases further, enhancing impaction and more central deposition of drug.

*After deposition:* Several factors can affect drug absorption and clearance from the respiratory tract following deposition. The mucociliary transport system, which covers the airways down to the terminal bronchioles, may decrease absorption. Either particles are trapped in mucus and transported to the oropharynx, or drug transport is affected by binding to mucus compounds. Absorption from the lung epithelium extending from the ciliated cells of the airways is also important. Aerosolized drug administered to the respiratory tract usually needs to cross the epithelial barrier to enter lung tissue for a topical effect, or the circulation for a systemic effect. Although pulmonary epithelium has a high resistance to movement of water and compounds that are not lipid-soluble, diffusion of poorly lipid-soluble compounds is often more rapid than expected, suggesting aqueous pores may aid pulmonary epithelial diffusion. Certain drugs such as sodium cromoglycate are known to undergo carrier-mediated transport. Enhancement of drug uptake by altered paracellular or transcellular (vesicular) transport is an exciting new area of research.

### Drug Delivery Devices

Unfortunately, many aerosolized drugs are marketed and trials undertaken with little information available to the clinician on drug output from various drug delivery devices. Such information is essential to optimize the therapeutic effect, and to avoid misinterpretation of results from clinical trials. The following examples of drug delivery, via inhalers with spacer device attachments and by nebulization, illustrate how basic information on the delivery method used can profoundly affect the dose of drug delivered to the patient.

*Metered dose inhalers and spacer devices:* Metered dose inhalers (MDI) cannot be recommended for use alone to deliver aerosols to children because of problems with co-ordination. Successful drug delivery from a metered dose inhaler requires co-ordination of actuation and inhalation, as the dose is best released at the beginning of a slow deep inspiration. The aerosol cloud they deliver contains micronized drug suspended in propellants and surfactant. Droplet size is 20–40 μm at the MDI orifice and the speed is approximately 30 m/s, decreasing to 3–4 m/s within a few centimetres. Droplet size decreases rapidly due to propellent evaporation. Of the particles delivered directly from a MDI, 80–90% cannot follow the inspired air stream and strike the oropharynx.

Spacer devices provide a reservoir of aerosol from which the child can inhale, with no need to co-ordinate inspiration and MDI actuation. Within the spacer device, the velocity of aerosol particles is reduced and surrounding propellants evaporate, leading to reduction in particle size. Drug delivery to the lung is equal to, or better than, when an MDI is used alone, and oropharangeal deposition of drug is markedly reduced. For instance, only 0.7 mg of a 5 mg dose of sodium cromoglycate is available for inhalation after actuation into a 750 ml spacer, yet slightly more drug is available from the spacer in particles likely to reach the lower airways. In the case of inhaled steroids, use of a large volume spacer has been shown to decrease suppression of the hypothalamic-pituitary-adrenal axis.[3,4]

The method of use and physical characteristics of the spacer may have profound effects on the amount of drug delivered.

Multiple actuations of drug into a spacer prior to inhalation should be avoided as drug output per actuation is considerably reduced. For example, if five actuations of a beclomethasone dipropionate MDI (250 μg/actuation, becloforte) are placed into a spacer and then inspired, the dose to patient is the same as if one actuation was placed into the spacer and inhaled.[5]

Drug should be inhaled immediately following actuation, as up to 50% of drug available for inhalation may deposit on the spacer walls within 10 s.

Using certain drugs, such as sodium cromoglycate (5 mg/actuation), twice as much drug is available for inhalation when a large volume spacer is used, as opposed to popular small volume spacers.

When generated, aerosols are usually highly charged. By coating the walls of polycarbonate spacer devices with an antistatic lining, which reduces electrostatic attraction, drug output may be considerably increased.[2]

*Nebulized drug delivery:* Only 10% of the inhaled dose reaches the lower airways in adults. The amount young children and infants inhale is unknown. The following examples illustrate the importance of a basic knowledge of nebulizer drug delivery.

The amount of nebulized drug available for inhalation, including that deposited in the nose and upper airways, may be independent of the child's age after 6 months of age.[6] Adults and older children inspire aerosol from the nebulizer, and, as their inspiratory flow and volume is large, they also entrain surrounding air. The younger the child, the less room air is entrained, and the more concentrated the aerosol they inspire. Although the total dose of aerosol inhaled per breath in young children and adults may be similar, dose per kilogram of body weight will be much greater in infancy. It has been suggested that, on the basis of these findings, aerosols should be administered in a weight-corrected basis for children aged more than 6 months of age.

A closely fitting face mask increases drug delivery, as a mask held just a couple of centimetres away from the face may result in a marked decrease (up to 85%) in drug inspired.[7]

Increasing the flow of gas through the nebulizer from 4 to 8 l/min alters the rate of drug output, the gas volume in which it is distributed, the aerosol concentration, and the volume of aerosol available during the inspiratory phase.[7] The amount of drug contained in particles smaller than 5 μm (respirable particles) increases considerably with higher flows.[1]

*Clinical examples:* Inhalation of drug aerosols has been popularized by the immense success in asthma treatment. Many other drugs are now given in aerosol form, for example:

Sputum viscosity is markedly enhanced in patients with cystic fibrosis due to DNA released from leukocytes and other cells. An enzyme DNase is now available that, when given by nebulization, markedly reduces sputum viscosity by splicing DNA strands.[8]

Nebulized pentamidine has been successfully used as prophylaxis against *pneumocystis carinii* infection in patients with human immunodeficiency virus infection. Work on deposition is continuing as recurrence of pneumocystis at the lung apex has been recorded.[9]

Nebulized Edmonston-Zagreb strain vaccine resulted in an 86% seroconversion in a group of 5-month-old children, protecting against measles. In younger children, administration of the vaccine by aerosol has the theoretical advantage that antibodies lining the respiratory epithelium, predominantly IgA, are less likely to be acquired from the mother and to inhibit viral replication, than circulating maternally derived antibodies which are predominantly IgG.

### References

1. Clay MM, Pavier D, Newman SP, Clarke SW. Factors influencing the size distribution of aerosol from jet nebulisers. *Thorax* 1983; **38:** 755–759.
2. O'Callaghan C, Lynch J, Cant M, Robertson C. Improvement in sodium cromoglycate delivery from a spacer device by use of an antistatic lining, immediate inhalation, and avoiding multiple actuations of drug. *Thorax* 1993; **48:** 603–606.
3. Brown PH, Blundell G, Greening AP. Do large volume spacer devices reduce the systemic of high dose inhaled cortico-steroids? *Thorax* 1990; **45:** 736–739.
4. Prahl P, Gents D. Decreased adreno-cortical suppression utilising nebuhaler for inhalation of steroid aerosols. *Clin Allergy* 1987; **17:** 393–398.
5. O'Callaghan C, Cant M, Robertson C. Delivery of beclomethasone diproprionate from a spacer device. What dose is available for inhalation? *Thorax* 1994; **49:** 961–964.
6. Collis GG, Cole CH, Le Souef PN. Dilution of nebulised aerosols by air entrainment in children. *Lancet* 1990; **336:** 341–343.
7. Everard ML, Clarke AR, Milner AD. Drug delivery from jet nebulisers. *Arch Dis Child* 1992; **67:** 586–591.
8. Shak S, Capon DJ, Hellmiss R, Masters SA, Baker CL. Recombinant human DNase 1 reduces the viscosity of cystic fibrosis sputum. *Proc Natl Acad Sci USA* 1990; **87:** 9188–9192.
9. Shanley DJ, Luyckz BA, Haggerty MF, Murphy TF. Spontaneous pneumothorax in AIDS patients with recurrent *pneumocystic carinii* pneumonia despite aerosolised pentamadine prophylaxis. *Chest* 1991; **99:** 502–504.
10. Sabin AB, Flores AA, De Castro F. Successful immunisation of infants with and without maternal antibody by aerosolised measles vaccine. *JAMA* 1983; **249:** 2651–2662.
